# Femoral–tibial contact stresses on fixed rotational femur models

**DOI:** 10.3389/fsurg.2022.1016707

**Published:** 2023-01-06

**Authors:** Peizhi Yuwen, Weiyi Sun, Jialiang Guo, Wenli Chang, Ning Wei, Haicheng Wang, Kai Ding, Wei Chen, Yingze Zhang

**Affiliations:** ^1^ Orthopaedic Research Institute of Hebei Province, Hebei Medical University Third Affiliated Hospital, Shijiazhuang, China; ^2^ Department of Hand Surgery, Cangzhou Hospital of Integrated TCM-WM of Hebei, Cangzhou, China; ^3^ Department of Orthopedic Surgery, Chinese People's Liberation Army Joint Security Force 980th Hospital, Shijiazhuang, China; ^4^ Key Laboratory of Biomechanics of Hebei Province, the Third Hospital of Hebei Medical University, Shijiazhuang, China; ^5^ Trauma Emergency Center, the Third Hospital of Hebei Medical University, Shijiazhuang, China; ^6^ NHC Key Laboratory of Intelligent Orthopeadic Equipment, the Third Hospital of Hebei Medical University, Shijiazhuang, China

**Keywords:** biomechanics, contact pressure, knee, fracture, internal rotation, external rotation

## Abstract

**Objectives:**

This study aims to quantitatively evaluate the femoral–tibial contact pressure on the knee under certain malrotaional degrees.

**Methods:**

Femoral–tibial contact pressure was carried out on 14 fixed rotational knee models under 200/400/600 N vertical load using ultra-low-pressure sensitive film technology, rotation angles including neutral position (0°, anatomically reduced), 5°, 10°, and 15° internally and externally. Data were collected and analyzed with SPSS software.

**Results:**

There are significant statistical differences between the medial contact pressure among rotational deformities (including neutral position) (*P* < 0.01), the increase in the degree of fixed internal malrotation of the femur resulted in a linear increase in the medial femoral–tibial contact pressures (*P* < 0.05) under 200/400/600 N vertical load, while increase in the degree of fixed external malrotation resulted in a linear decrease (*P* < 0.05). Except the 200 N compression, we can't find significant differences in lateral contact pressures (*P* > 0.05). In the comparison of medial to lateral contact pressures, no statistically significant differences were found in neutral and 5° internal rotation under 200/400 N, neutral, 5° internal rotation, and 15° external rotation under 600 N. In contrast, medial contact pressures were higher than lateral at other angles (*P* < 0.05).

**Conclusion:**

Obvious contact pressure changes were observed in rotatory femur. Doctors should detect rotational deformity as much as possible during operation and perform anatomical reduction. For patients with residual rotational deformities, indication of osteotomy should not be too broad.

## Introduction

Rotational deformity is one of the most significant complications of femoral shaft fractures, and many studies have mentioned its high incidence (1–4). More than 15° torsion deformity alters gait mechanics and efficiency (5–8), as it completely disrupts the biomechanical relationship of hip and knee joint or both and changes the stroke and direction of related muscles. Patients might develop degenerative arthritis of the knee (KOA) and obvious local symptoms in the long term (9–14).

Femur osteotomy is a well-established surgical treatment for femoral torsion (6). Generally, a malrotation <10° is reckoned as a normal alteration, >15° is a true torsional deformity (1, 15, 16), and between 10° and 14° is considered a “gray area” (15–17). However, this conclusion comes from a large number of clinical observations, patient's subjective feelings, and different conclusions in the literature, so robust evidence is needed for surgical indications. We, therefore, bring this biomechanical study to understand the changes of the medial and lateral femoral–tibial contact pressures under different rotation angles and compressions and to provide theoretical support for the cutting angle of femoral osteotomy.

## Materials and methods

This study has been approved by the Institutional Review Board (IRB) of the Third Hospital of Hebei Medical University (2017-003-1). All cadavers were voluntarily donated and provided by the Department of Human Anatomy, Hebei Medical University. Consent for the storage and use of the bodies for research purposes was given by all body donors before death or by their next of kin.

Specimen preparation: Fourteen fresh-frozen cadaveric lower limbs with intact soft tissue were autopsied; the mean height of the donors was 171 cm (range, 163–181 cm), and average age was 55 years (range, 42–65 years). No history of surgery and gross knee deformities, i.e., hyperflexion, hyperextension, varus, and valgus were observed of all specimens. The passive joint motion was freely achieved. Subsequently x-ray examination showed that all inner structures were intact.

**Figure 1:**
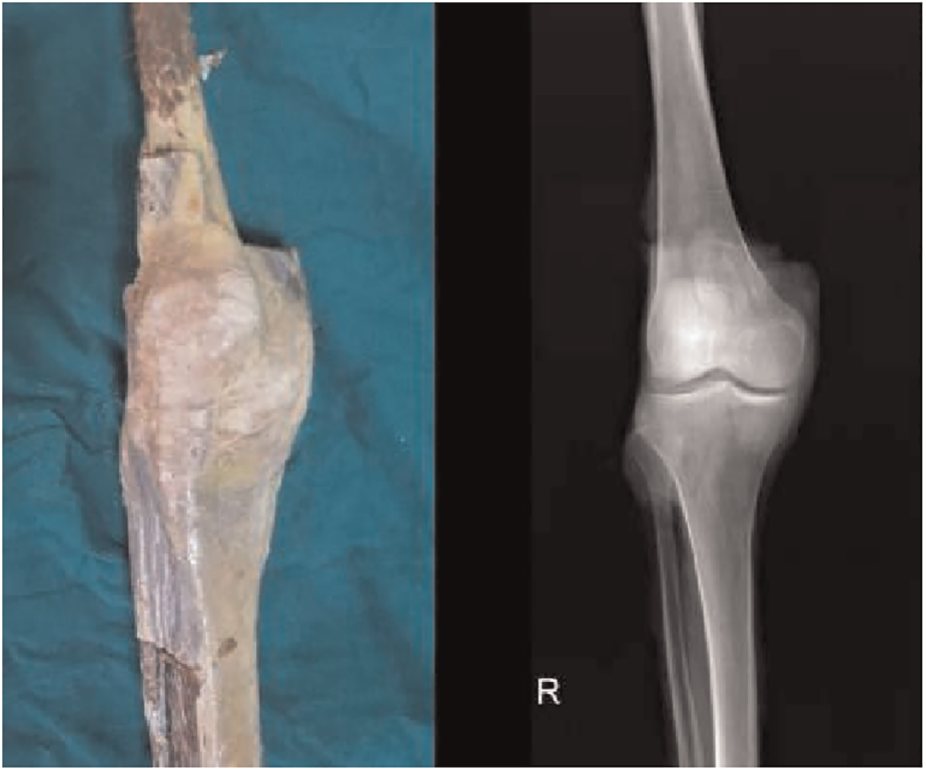
General photograph and x-ray of a cadaveric knee.

The muscular tissues are carefully excised, and the intact joint capsule and surrounding overlying ligaments are observed (Figure 1). A horizontal incision approximately 3–4 cm long is made along the level of the joint space on either side of the patellar ligament, close to the inferior border of the patella. The subcutaneous fat and capsule are separated and the joint space is exposed. Particular attention needs to be paid to checking the integrity of the anterior and posterior cruciate ligaments and meniscus, as the meniscus is a load-bearing structure that buffers pressure and affects the expansion (18). The lower limb mechanical force line is marked prior to the experiment (17).

Establishment of rotatory fixation models: The femoral shaft was sawn transversely at the distal one-third, rotated at a specific angle, and then both femoral stumps were fixed with plates and screws. Specific angles included neutral (0°, anatomically reduced), 5°, 10°, and 15° internal and external rotation. To facilitate retrofitting to the biomechanical machine, the distal femur and the proximal tibia and fibula were each retained at 25 cm.

**Figure 2:**
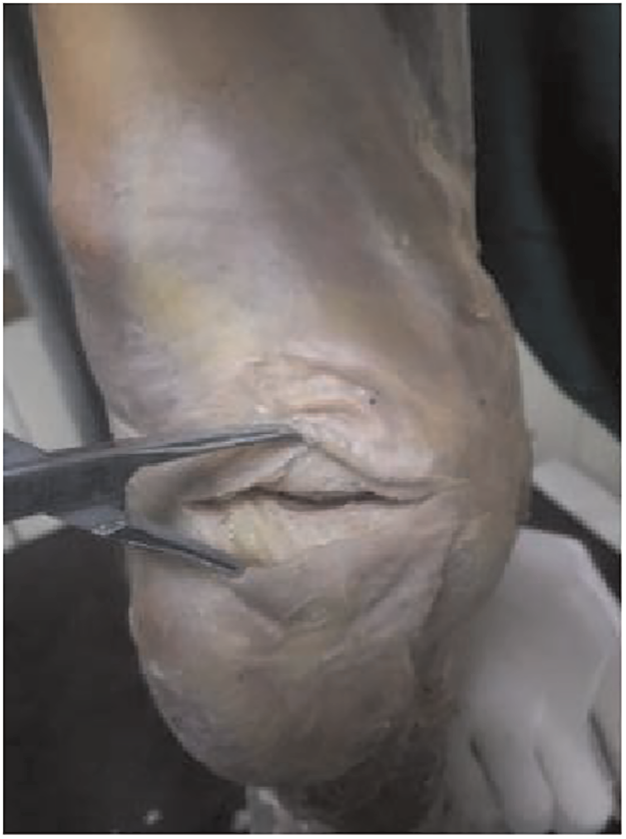
Two horizontal incisions about 3–4 cm long were made.

An ultra-low-pressure sensitive film (LLW type, Fujifilm Investment Co. Ltd. Japan) was used to measure the intra-articular femoral–tibial contact pressures. The room humidity was set at 35% RH and the temperature was 20 °C to ensure its sensitivity. Fuji films (0.5–2.5 MPa) were trimmed to fit the joint lacuna, in order to prevent contamination; then, they were wrapped and sealed with a thin polyethylene sheet (total thickness, approximately 250 pm). After finishing, two wrapped Fuji films were carefully inserted into the space beneath the medial and lateral meniscus through the anterior incision, ensuring that they were fully accessed, suturing the capsule tightly; any leaking, bent, or broken seal pocket meant failure (19) (Figure 2). Before insertion, indentations are made with a hemostat to distinguish the anterior and posterior side of the Fuji films.

**Figure 3:**
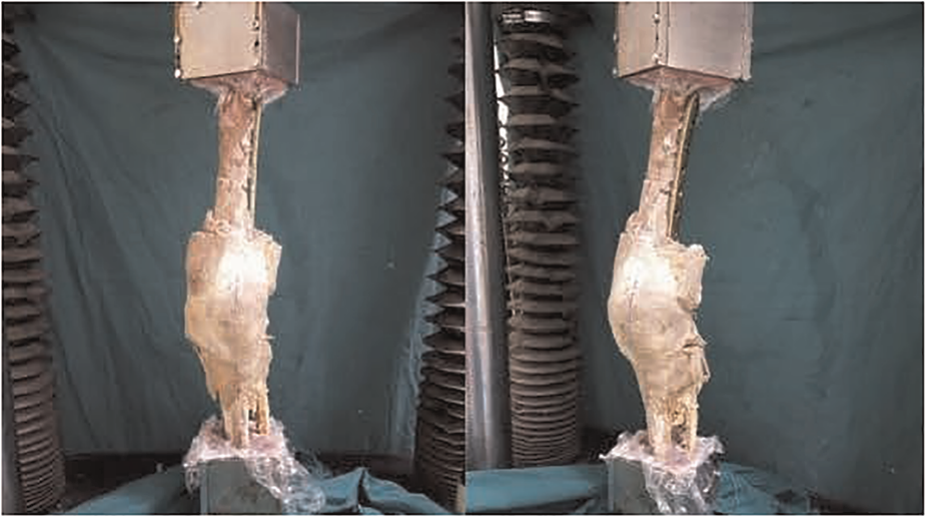
Model of external rotatory deformity. The specimens were assembled to the BOSE Electroforce 3520-AT biomechanical testing machine.

**Figure 4:**
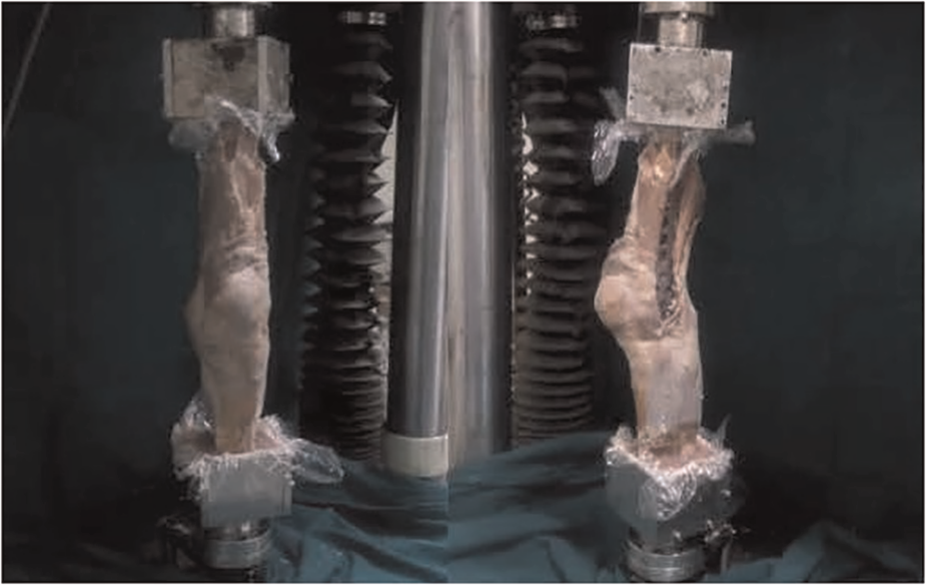
Model of internal rotatory deformity. The specimens were assembled to the BOSE Electroforce 3520-AT biomechanical testing machine.

Assembling models: The models were rigidly clamped in a self-made iron square trough in anatomic position, and two ends were embedded in a mixture of denture base resin (type II self-setting denture base powder) and denture base resin liquid (type II self-setting denture base water) (Figures 3, 4). The completed fixed model was then transferred and assembled into the biomechanical test machine (Electroforce 3520-AT, Bose company, United States).

To eliminate creep, biomechanical machine was started and the test bench was loaded with a pressure up 200 N at a speed of 10N/s. After stabilization, axial compression up to 400 N was applied to each knee and upheld for 2 min; the Fuji films were took out and unloaded. Pressure values were read through the FPD-305E density meter and the FPD-306E pressure converter. Experiments were carried out as above with 200 and 600 N.

Measurements were repeated three times for each specimen to eliminate interobserver variation. Saline was continuously sprayed on the specimens during the experiment to prevent drying from affecting the accuracy of the experimental data.

### Statistical analysis

Experimental data were collated and calculated using SPSS 21.0 software (SPSS, Chicago, IL, United States). Normality was verified using the Shapiro–Wilk test and expressed as $\bar{x}\pm s$. We used *t*-tests for two independent samples to obtain differences between the medial and lateral groups, and the Student–Newman–Keuls test was used for pairwise comparisons between multiple sample measures. The Levene test was used to test for consistency of variance, and analysis of variance (ANOVA) was performed on randomized groups of zones. Data that did not conform to normality were expressed as median (quartiles), and the Mann–Whitney *U* test was used to obtain differences between the inner and outer groups. The Kruskal–Wallis *H*-test was used for the random block group; *P* < 0.05 indicated significance.

## Results

**Figure 5:**
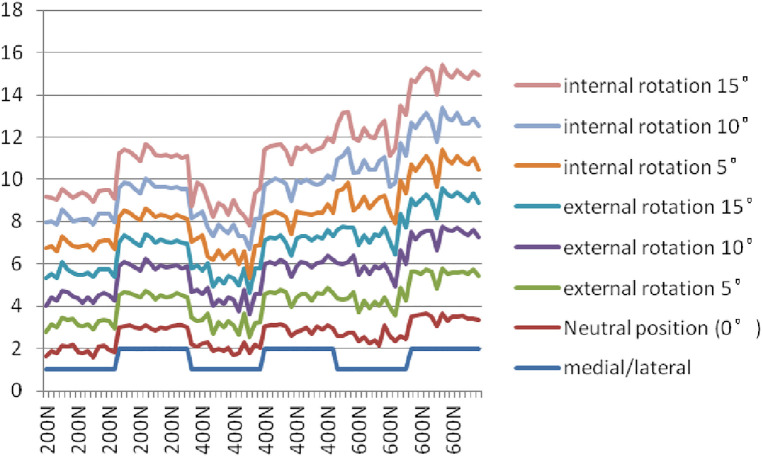
Medial and lateral femur-tibia contact pressure at 200/400/600 N compressions.

Tables 1, 2 show the calculated contact pressures in neutral position (0°, anatomically reduced)5°, 10°, and 15° of internal and external rotation under 200, 400, and 600 N vertical compressions (Figure 5).

**Table 1:** Value of medial femoral–tibial contact compression at various rotatory angles (MPa).

Medial contact pressure	200 N	400 N	600 N	*F*	*P*
Rotation deformity	Average contact stresses (MPa)	Average contact stresses (MPa)	Average contact stresses (MPa)		
Neutral position (0°)	1.012 ± 0.083	0.952 ± 0.168	1.466 ± 0.163	32.590	0.000
External rotation 5°	1.507 ± 0.065	1.601 ± 0.093	2.097 ± 0.128	26.462	0.000
External rotation 10°	1.388 ± 0.064	1.472 ± 0.075	1.885 ± 0.139	80.828	0.000
External rotation 15°	1.187 ± 0.057	1.172 ± 0.096	1.638 ± 0.086	68.443	0.000
Internal rotation 5°	1.197 ± 0.028	1.151 ± 0.082	1.667 ± 0.088	13.942	0.000
Internal rotation 10°	1.365 ± 0.049	1.493 ± 0.085	1.998 ± 0.080	46.916	0.000
Internal rotation 15°	1.540 ± 0.060	1.645 ± 0.088	2.159 ± 0.124	46.051	0.000
*F*	141.197	92.114	66.795		
*P*	0.000*	0.000*	0.000*		

* *P* < 0.01.

**Table 2:** Value of lateral femoral–tibial contact stresses at various rotatory angles (MPa).

Lateral contact pressure	200 N	400 N	600 N	*F*	*P*
Rotation deformity	Average contact stresses (MPa)	Average contact stresses (MPa)	Average contact stresses (MPa)		
Neutral position (0°)	0.917 ± 0.191	1.023 ± 0.208	1.535 ± 0.247	53.836	0.000
External rotation 5°	1.243 ± 0.062	1.141 ± 0.208	1.670 ± 0.278	144.667	0.000
External rotation 10°	1.263 (0.064)	1.209 ± 0.121	1.671 (0.145)	102.806	0.000
External rotation 15°	1.183 ± 0.096	1.067 (0.206)	1.563 ± 0.134	148.644	0.000
Internal rotation 5°	1.314 ± 0.056	1.098 ± 0.333	1.636 (0.320)	223.312	0.000
Internal rotation 10°	1.245 ± 0.027	1.221 (0.225)	1.669 (0.249)	293.167	0.000
Internal rotation 15°	1.121 ± 0.077	1.114 ± 0.243	1.662 ± 0.158	173.612	0.000
*F*	57.007	9.967	9.003		
*P*	0.000*	0.126	0.173		

* *P* < 0.01.

The contact pressures in neutral position are the lowest on both medial and lateral sides compared to other angles under any compressions.

The increase in the degree of fixed internal malrotation of the femur resulted in a linear increase in the medial femoral–tibial contact pressures (*P* < 0.05) under any compressions, while an increase in the degree of fixed external malrotation resulted in a linear decrease (*P* < 0.05). There are significant statistical differences between the medial contact pressure among rotational deformities (including neutral position) (*P* < 0.01); comparisons in pairs were all significant (*P* < 0.05).

Except the 200 N compression, we could not find significant differences in lateral contact pressure among malrotaional angels (*P* > 0.05).

In the comparison of medial to lateral contact pressures, no statistically significant differences were found in neutral and 5° internal rotation under 200/400 N, and neutral, 5° internal rotation, and 15° external rotation under 600 N. In contrast, medial contact pressures were higher than lateral at other angles (*P* < 0.05) (Tables 3–5).

**Table 3:** Comparison of contact stresses between medial and lateral femoral–tibial side under 200 N.

	Neutral position (0°)	External rotation 5°	External rotation 10°	External rotation 15°	Internal rotation 5°	Internal rotation 10°	Internal rotation 15°
*t/Z*	−1.715	−10.967	−4.413	−0.122	6.963	−8.067	−16.136
*P*	0.104	0.000*	0.000*	0.904	0.000*	0.000*	0.000*

* *P* < 0.05.

**Table 4:** Comparison of contact stresses between medial and lateral femoral–tibial side under 400 N.

	Neutral position (0°)	External rotation 5°	External rotation 10°	External rotation 15°	Internal rotation 5°	Internal rotation 10°	Internal rotation 15°
*t/Z*	0.998	−7.525	−6.909	−2.160	−0.578	−4.251	−7.673
*P*	0.327	0.000*	0.000*	0.031*	0.572	0.000*	0.000*

* *P* < 0.05.

**Table 5:** Comparison of contact stresses between medial and lateral femoral–tibial side under 600 N.

	Neutral position (0°)	External rotation 5°	External rotation 10°	External rotation 15°	Internal rotation 5°	Internal rotation 10°	Internal rotation 15°
*t/Z*	0.865	−5.227	−3.771	−1.767	−0.092	−4.412	−8.593
*P*	0.395	0.000*	0.000*	0.091	0.946	0.000*	0.000*

* *P* < 0.05.

## Discussion

Residual rotational misalignment of the femur remains a Gordian knot (20, 21). Winquist et al. (3) conducted a study on 520 femoral shaft fractures and noticed that 8% of patients had postoperative external rotation deformities of more than 10°. Bråten et al. (1) in 1993 found that 19% of patients had more than 15° malalignment after intramedullary nailing nailing of femoral fractures. Sennerich et al. (2) reported that 40% patients had more than 10° of rotational malalignment and 16% had more than 20°. Hufner et al. (22) documented that 22% of 82 patients had a rotation deformity more than 15° after intramedullary nails. Others (5, 23, 24) reported an even higher incidence.

In our biomechanical study, the contact pressures in neutral position are the lowest on both medial and lateral sides compared to other angles under any compressions. Indicating that any rotation deformity will result into the contact pressure increasing, which is consistent with the conclusion of the study by Thorp et al. (25), the contact pressure on the medial compartment was significantly higher in patients with knee osteoarthritis than normal ones during walking. It is not difficult to conclude that rotation deformity is one of the risk factors of knee arthritis. Changes in intra-articular pressures and asymmetric weight-bearing during movement exceed the elastic potential capacity of the cartilage and subchondral bone. In addition, preexisting axial pressures are partially converted into shear forces due to rotational deformities, causing a local biomechanical chain reaction (26, 27).

We also found that the medial contact pressure is close to the lateral side at 0° and 5° internal rotation; a possible explanation is that the human body has certain adaptability, trying to balance the increased pressure. While exceeding 5°, the medial contact pressures were higher than the lateral side, the human body begins to lose balance and the medial compartment bears the brunt. Our findings coincide with those of Foroughi et al. (28) that changes in the medial compartment are the most pronounced in degenerative arthritis, occurring 10 times more frequently than the lateral compartment.

Poor reduction and postoperative malalignment is the main cause of rotational deformities. Early detection during operation can help surgeons to improve the quality of fracture reduction, but once rotational deformity is identified postoperatively, osteotomy is inevitable. However, the indications are still unclear. Lee et al. (29) argue that as long as the deformity is obvious, it can be corrected by osteotomy. Piper et al. (30) believed that internal rotation exceeding 10° can be corrected by osteotomy. Citak et al. (31) found that indication could be relaxed to 15°, as hideously torsion can severely affect activities and even lower limb function abnormalities. Other authors (2, 32) have concluded that torsional deformity of less than 20° is not usually a barrier. We found that contact pressure on the medial side decreased with the aggravation of external rotation and increased with the aggravation of internal rotation, but both were higher than the neutral position. There is no significant difference in lateral contact pressure at all malrotaional angels. Therefore, we recommend that doctors should give more attention to internal rotation than external rotation. From our point of view, the indications for osteotomy should not be too broad, although some patients can tolerate certain degree of torsional alignment.

Certain limitations are evident in this study. First, this was an *in vitro* study, which is not fully representative of the muscle dynamics of a normal human being. Second, our study fixed the models along the anatomical axis, which may increase the femoral–tibial contact pressure on the medial side. Third, vertical compression is definitely small to simulate human beings in walking or running activities, and in future, lager pressure experiments are expected.

## Data Availability

The original contributions presented in the study are included in the article/Supplementary Material, further inquiries can be directed to the corresponding author.
